# Navigating the care between two distinct cultures: a qualitative study of the experiences of Arabic-speaking immigrants in Norwegian hospitals

**DOI:** 10.1186/s12913-022-07833-6

**Published:** 2022-03-26

**Authors:** Tariq Alkhaled, Gudrun Rohde, Birgit Lie, Berit Johannessen

**Affiliations:** 1 Department of Health and Nursing, Faculty of Health and Sport Sciences, University of Agder, Postbox 422, 4604 Kristiansand, Norway; 2 Department of Clinical Research, Sorlandet Hospital, Kristiansand, Norway

**Keywords:** Cultural competence, Trans-cultural health care, Foreign-born patients, Ethnically-diverse patients, Linguistic problems, Norwegian health service

## Abstract

**Background:**

During the past decades, there has been an increase in the number of immigrants to European and Scandinavian countries. This has challenged the health-care systems, which cater to the needs of patients despite their cultural and linguistic barriers, in these countries. Most studies on this topic have focused on the perspectives of health-care providers in delivering their service. The purpose of this qualitative study was to explore how hospitalized Arabic-speaking patients experienced their interaction with the Norwegian health-care system.

**Methods:**

In-depth interviews with 20 participants and researcher’s participant observation were conducted to explore the idiosyncratic details and ascribed meanings that foreign-born patients attach to their everyday experience of the Norwegian health-care system. Thematic analysis was performed on the transcribed and translated versions of the in-depth interviews.

**Results:**

The findings of this study indicated three interrelated core themes. Firstly, there exist challenges in understanding and being understood because of linguistic and cultural differences of newly migrated patients. Secondly, some patients missed the holistic and direct care available in their home countries. Finally, patients were satisfied with the Norwegian health-care system because of its compassion, care, and respect toward them as well as advanced health-care equipment.

**Conclusion:**

Arabic-speaking patients in Norwegian hospitals experienced long waiting times and linguistic problems. Hence, a better and specialized interpreter service may resolve problems emanating from communication within the system and the subsequent delays in treatment. Compassionate care and the feeling of respect and love is the core strength of the Norwegian system as perceived by the patients.

## Background

Over the last decade, there has been a steady increase of multicultural patients in the Norwegian health-care system [1]. The need to cater to the cultural diversity among patients is founded in the important principle of equitable access to health-care services. Recent studies have debated the trade-off between the rights of foreign patients and their demands from sociolegal and moral standpoints [2, 3]. Nevertheless, there exists a paucity of literature on the perceptions and lived experiences of immigrant patients regarding the access and likely linguistic and cultural barriers to health care.

Previous research showed that the migrant communities in Norway lag behind the indigenous population in terms of access to health care despite the generous nature of the welfare system [4]. Even though Norway has one of the highest per capita expenditures on health and the highest availability of physicians and nurses per 100,000 individuals, equitable access to health care is still a part of the debate on health policies. Norway has a tax-funded public health-care system, and all Norwegian residents have equal access to health-care services. All persons registered in Norway have the right to choose their own general practitioner. The Norwegian health-care system is semi-decentralized, which means that the state is in charge of specialist care while municipalities are in charge of primary care including general physicians [5, 6]. Immigrants who do not speak Norwegian have the right to a free interpreter service during medical appointments, as stated in the National Strategy on Immigrants’ Health [7].

Some studies investigated various dimensions of unmet needs and experiences of diverse populations within advanced western health-care systems, such as that in Norway. These studies include consideration of the cultural competency of the nursing staff [2, 8, 9], inclusionary and exclusionary organizational practices [1], cultural diversity [10],comparative experiences between the indigenous people and immigrants [11], normative and ethical debates regarding immigrant patients [3, 12, 13] and familial involvement [14–16].

Most previous studies tended to conceptualize the problem from the perspective of service providers, and reports on phenomenological experiences of the cultural diversity from the patients’ perspectives, especially Muslim or Arabic-speaking patients, are sporadic [17, 18]. In this context, Abebe (2010) [13] analyzed various policy documents and reported that non-Western immigrants were more susceptible to health conditions, such as chronic diseases, mental health, teeth-related problems, and lifestyle issues with health implications.

Arabic-speaking patients among many culturally diverse patients are likely to have a spiritual, cultural, or religious connotation attached to the hospitalization experience, often requiring their families and friends to visit them [19, 20]. Until now, how a patient’s cultural, communicational, and religious characteristics interact with their utilization of a health-care service has not been well investigated [21]. Moreover, linguistic issues are important because they accentuate and amplify other cultural barriers, such as values, norms, and gender-related issues, through a feeling of miscommunication and exclusion [22–26].

Internationally, the intersection of health care with the cultural and linguistic adaptation of immigrants has brought various specific issues to the surface. These recurrent themes include the experience of trauma by the immigrants [27], sexual health [28], and complementary and alternative medicine (CAM). CAM is a set of diverse diagnostic and therapeutic procedures, as well as a range of natural products used for the treatment of patients, derived from previously known traditional methods and enriched with modern scientific knowledge [29, 30]. CAM is not generally available in the public health-care service in Norway, so that people who want to be treated with CAM must pay for this treatment in the private market [31].

While cultural competence and linguistic skills are important both for the nursing staff and health-care providers to perform their duties [32], they have not been investigated thoroughly from the patient’s perspective. The holistic cultural needs of the patients and their cultural care have recently acquired some prominence in the literature; for instance, regarding the issue of female patients with genital mutilation accessing Norwegian health care [33, 34].

Hence, the aim of the present study is to explore how hospitalized Arabic-speaking patients experienced their interactions with the Norwegian health-care system.

## Methods

### Study design

To explore how hospitalized Arabic-speaking patients experienced their interactions with the Norwegian health-care system, we chose a hermeneutic phenomenological approach using in-depth qualitative interviews.

### Recruitment process

The head nurses at five hospitals were contacted and informed about the study. They distributed the written information about the study and the interview guide to the nursing staff. Written detailed information and informed consent forms in Arabic were circulated among to those patients who wanted to participate. The written information included a description of the aim of the study, the aspects of voluntary participation, the possibility to withdraw at any time, and the confidentiality of the handling and presentation of data. Patients who showed an interest in participating were asked for their informed consent and their telephone number or E-mail address. They were then contacted by the researcher to decide the proper place and time for the interviews to take place.

### Participants

Inclusion criteria for participation in the interview were Arabic-speaking adults (aged over 18 years) who had been hospitalized for 2 days or more in a Norwegian hospital. In total, 20 patients consented to participate in the study. The participants were from six different Arabic countries (Iraq, Syria, Egypt, Yemen, Morocco, and Palestine). The sample was purposive and comprised 18 men and two women. The participants were aged 26–61 years (average age, 50 years). Most participants (*n* = 16) had a low level of education while four had a university education (Table 1). Nine participants had limited to no skills at speaking Norwegian, 10 had an average–good grasp of the language, and only one described their linguistic ability as fluent. The length of their stay in Norway ranged from 1.5 years to 22 years.

**Table 1:** Background characteristics of the participants

Participant	Sex	Age (years)	Number of years living in Norway	Education	Command of Norwegian as defined by participants
R1	Male	54	3.5	Bachelor	None
R2	Male	61	5	Bachelor	Limited
R3	Male	52	1.5	High school	None
R4	Male	60	4	Primary school	Limited
R5	Female	45	4	Primary school	Limited
R6	Male	46	4	Primary school	Limited
R7	Male	35	4	Secondary school	Average
R8	Male	43	13	Secondary school	Fluent
R9	Female	26	5	Graduate	Good
R10	Male	30	5	High school	Average
R11	Male	50	3	Primary school	Limited
R12	Male	32	5	Primary school	Average
R13	Male	58	5	Secondary school	Average
R14	Male	58	13	Primary school	Limited
R15	Male	43	4	PhD	Good
R16	Male	61	22	Primary school	Average
R17	Male	58	3	Secondary school	Limited
R18	Male	38	6	Primary school	Good
R19	Male	41	6	Primary school	Average
R20	Male	43	10	Secondary school	Good
Range		26–61	1–22	Primary to PhD	None to Very Good

### Data collection

Data were collected over a 9-month consecutive period during 2020–2021. Interviews were conducted in Arabic. The interview guide addressed the themes of linguistic competencies during communication, cultural issues such as values and beliefs, experience of pain, the role of the patient’s family, meals, and their experience in dealing with the Norwegian health-care system. The interview guide was validated by the scrutiny of three experts in the field. The guide was translated into Arabic and pretested on two Arabic-speaking individuals. Some required modifications were made. Due to the aggravated concern over Covid-19, we were not allowed to conduct the interviews in the hospitals. According to the participants’ wishes, all interviews were conducted in their homes. Each interview lasted 40–60 min. The interviews were transcribed in Arabic and subsequently translated into English by the researcher.

### The research team

The research team consisted of four researchers from different backgrounds. The first author (TA) is an Arabic-speaking Syrian nurse and PhD candidate who has been living in Norway for the last 6 years.

The other three researchers in the team are Norwegians with a medical or nursing background and an immigrant research focus.

### Analyses

The data were analyzed using thematic analysis, which was based on a combination of two sets of guidelines because of their flexibility and practical nature. The general guidelines and principles of hermeneutics as proposed by Van Manan [35] were combined with an analytical and step-by-step approach proposed by Braun and Clarke [36], which includes the following steps: (1) familiarizing with the data, (2) creating initial codes based on significant statements, (3) organizing the codes under themes, (4) reviewing the themes for overlaps and contradictions, (5) defining and naming the themes, and (6) reporting the findings.

Three of the researchers (TA, BJ, GR) read and re-read participant interview statements before creating initial line-by-line codes based on statement significance. These initial codes were grouped to form general themes, which were then checked against the participants’ original transcripts and reviewed for any overlaps or contradictions before the final theme labels were created. The last stage of the analysis involved the write-up of the results, which allowed the researcher to share the participants’ experiences.

## Results

**Fig. 1:**
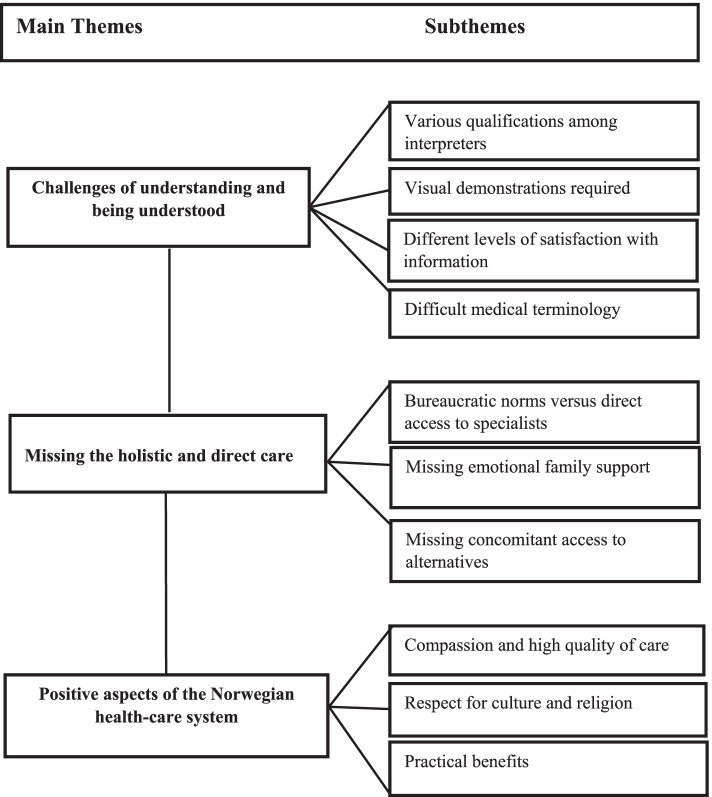
Main themes and subthemes as reflected in the narratives of Arabic-speaking patients

This study aimed to explore how hospitalized Arabic-speaking patients experienced their interaction with the Norwegian health-care system. Subsequent data analysis produced the following main themes and subthemes (Fig. 1).

### Challenges in understanding and being understood

The participants described that there could be barriers to effective communication about health issues, which could affect their ability to understand their doctors or for the doctors to understand them. Therefore, the participants employed different methods to ensure that they could effectively and reciprocally interact with the health-care system and its actors to obtain and furnish the essential information about their disease and treatment. These methods included using an interpreter when they had limited to no command of Norwegian, an interpreter specifically for understanding specialist terminology, English as a bridge to communication, and, in two cases, a video to convey information visually. None of the participant mention confidentiality issues in the presence of an interpreter. The majority would not prefer that one of their family members be an interpreter.

In particular, many participants had used an interpreter if they had limited to no command of Norwegian or when a specialist area of medicine needed to be discussed. One participant stated: *“My health situation is quite complicated, so it was necessary to use a specialized interpreter.” (Participant 14).*

Some participants received interpretation services from people related to medical institutions. These “specialists” were perceived as better mediators with the ability to bridge the cultural and linguistic differences. In addition, translation by no-specialists was often blamed for delays, misunderstandings, and a sense of aloofness.

*Once, I had an appointment with the doctor, I was asked if I want to have surgery; I inquired if there were any dangers and* complications…*A few weeks later I had an appointment with the GP, and I was surprised when he told me that I refused to have the surgery. That means there was a misunderstanding, or the interpreter was not able to convey the right idea. I've suffered a lot because the interpreters were not able to convey the right information to the doctors. (Participant 3).*

In contrast, one participant who had limited command of the language described how using an interpreter with a medical background from his own culture enabled clear communication within the patient–doctor interaction. In general, participants described how indirect communication (i.e., via an interpreter) could be a “challenge” and expressed a strong preference for direct communication between themselves and their doctor that could allow them to better participate in making decisions about their care. To help with direct communication, some participants had used English as a mediating language to bridge a gap in understanding between the Norwegian and Arabic languages. Two participants experienced some difficulties in understanding the procedure, and the doctors used visual help and images to facilitate the understanding of surgical and anatomical information.

Although there could be some issues with understanding medical terminology, most participants believed that owing to the different opportunities for sharing knowledge, they did receive the information they needed about their health care. As a male with only primary school education and little command of the Norwegian language who had used an interpreter explained, *“the information I received was good and I got all the information I wanted, especially concerning the surgery.” (Participant 11)*.

Similarly, another participant described how, despite difficult medical terms, with the use of a translator, he was able to understand all but 5% of the information and felt he received complete information about his care.

*Yes, the doctor provided me with full information about the condition and the treatment plan. The information was good…Only about 5% I couldn't understand, the reason behind that is the translation and the difficult medical terms they used. (Participant 17).*

It was noticed that problems of communication amplify the perception of cultural misunderstandings. Most problematic experiences were narrated by participants who had spent four or fewer years in Norway.

### Missing the holistic and direct care

This theme describes the participants’ experiences of how they missed their home culture and how this culture appeared to some extent to clash with aspects within the Norwegian health-care system. In particular, they spoke about their satisfaction with the direct access to specialists in their home country compared with their dissatisfaction with the referral process via a general practitioner (GP) in Norway; the lack of alternative treatment, such as that using herbs; the lack of family support; and experiences of non-halal meals at Norwegian hospitals.

In the participants’ home countries, the health-care system differed in that they could select a specialist doctor within the hospital and could directly visit the doctor, without any waiting times or a need for a referral from a GP, as expressed by a participant*: “I would be able to see the doctor directly without an appointment, and I would be able to decide which doctor I would see.” (Participant 4)*. In contrast, in Norway, the GP acts as a gatekeeper to specialist care. This contrast in the different health-care systems appeared to explain much of the dissatisfaction that the participants experienced with the Norwegian health-care system. In particular, the participants described a general dissatisfaction with long waiting times and a view that GPs have limited expertise.

*Because the health system here is different from the one in my country, so it is normal to have confusion in dealing with it. For example, getting sick here, I must see the GP, even though I know I need to see a cardiologist…Why can’t I see the specialist directly? (Participant 15).*

Concerning the experience of waiting times, some participants also expressed dissatisfaction with time restrictions on their appointment with the GP or the specialist, as explained by one of the participants, *“Sometimes it takes a long time for the patient to be diagnosed and get the treatment. That’s because of the bureaucracy and the long waiting time to see a specialist.” (Participant 15).* Similarly, another participant explained how he had to wait for long time until he got help, “*I needed surgery, but it was delayed for a long time because I didn’t get residency…I waited a long time until I got help and started treatment.” (Participant 6).*

Some participants also mentioned a wrong diagnosis or procedural mistake as explained by one of them.

*Until this day, I haven’t received any detailed information related to the medical mistake that happened to me, no clear answers, or explanations. After the medical mistake that happened to me, I have no confidence at all. And I told that to the physician. (Participant 1).*

Usually, the GP is the first point of contact with the services of the health-care system. The ineffective communication between the patient and the GP ends up being associated with the perception of imprudence, inexperience, and lack of knowledge in the GP by the patient. Therefore, most participants tended to present their GP in a negative way. When they were asked about something that they wanted to change about the system, they were most likely to say their GP. Some participants described that their condition worsened because their GP was not able to treat their condition.

*I got sick and went to the GP many times. Every time I went there, the GP told me that’s normal and there’s nothing abnormal. My health situation was getting worse. (Participant 12)*

Some participants also experienced GPs who used the internet in their consultations to find health information. This was perceived to be unacceptable by the participants and eroded their faith in the doctor’s ability to treat them.

One cultural belief in Arabic-speaking patients was the role of herbs as an alternative treatment for health conditions. Most participants had tried using herbs in their health care. Moreover, some participants believed in the use of herbs in the treatment of various ailments and used them if they were available in Norway. However, the participants made no extra efforts to avail herbs if they were not available or approved for use.

*I use herbs that are tried and tested, and I feel I can trust them. But I have the most confidence in an experienced doctor; I will ask them and follow their advice related to that. (Participant 19).*

Two participants also mentioned the role of God in their health care; i.e., that God had control over their healing process. Regarding the role of medicine, herbs, and God within the healing process, the participants mentioned that God will decide whether treatments from medicine or herbs would work.

*I believe in disease and healing from God, but we must take into account the reasons. I have to seek help and take the medication, and after that, ask God for healing. Some herbs are useful for some diseases. If I hear about a proven herb or that somebody tried it and found it useful, I will try it. (Participant 8).*

Most participants expressed that they wanted their family members to be present during the period of hospitalization. The participants held culturally different meanings for the presence of families in the hospital. For some, family presence was deemed essential—they needed their family to provide important emotional and practical support while in the hospital. For example, a participant explained that his family was performing the role of an interpreter: *“I don’t speak Norwegian and was hospitalized twice. At both times, there was no interpreter. I needed someone to interpret to the doctors.” (Participant 3)*. Some participants believed that family support was essential for providing personal care or necessary emotional support.

*I feel relaxed and safe to have someone from my family next to me. And it is essential to have them help me with my personal needs. (Participant 16)*

Another participant explained:

*We are emotional people who need someone to be next to them in case of illness…I got a lot of visitors when I was hospitalized, so the staff there were surprised. One of them said to me that, we lack the family relationships and emotions your community has. Feeling the presence of your family around you and their care in a time of distress is important. (Participant 17).*

Some participants found it difficult or uncomfortable to accept support for personal needs from the nurses as well as to ask them for help and reported psychological reasons for needing their family around. An altruistic reason for asking family to help with personal needs was considering the nurse’s role to be purely medical. One participant described cultural reasons for his need for familial support interpreted by his use of *“we are emotional people”* and his experience with the nurse describing his *“community.”*

*For me, it is difficult to ask the nurse for help. In my opinion, sick people need to be accompanied by a family member to feel safe and comfortable. It is a kind of psychological support. (Participant 4).*

In contrast, another participant mentioned*, “The nurse’s job is not to help with personal needs, to bring me a glass of water, or to help me go to the bathroom. Her job is to give me medicine or change bandages, something like that.” (Participant 9).*

When the participants were asked about how they expressed pain, many said that they were unable to express pain in front of their families. However, they were able to express pain to nursing staff.

*I try to never show my family that I have pain, especially the children, so they don’t worry about me. I try not to tell my wife either, because she calls the ambulance at once. But at the hospital, I told them right away. (Participant 12).*

The food served in the hospital was different from what the participants would get in their home culture, and they had mixed experiences of whether the hospital could provide the food that they needed. Some participants believed that generally *“The meals in the hospital were good”* and that alternative and abundant choices were available. However, some participants mentioned that halal food was not available.

*It was not good. The type of meals provided in the hospital is completely different from the food I’m used to. For example, sometimes there was pork and fish, and I don’t eat that, so I was saying I’m not hungry, and I didn’t ask for anything else. (Participant 9).*

Another participant mentioned.

*Honestly, I never ate in the hospital. I was there for two days; I only drank water in the hospital. Almost all the food provided in the hospital was not suitable for me. If I stay there longer, I'll definitely eat, and I would tell them about the foods I don't eat. (Participant 10).*

However, some participants were happy to order vegetarian options to avoid non-halal meat. Other participants were able to get the meals that they wanted from their wives.

### Positive aspects of the Norwegian health-care system

The positive aspects of the health-care system tended to focus on general satisfaction with the treatment received in Norwegian hospitals; experiences of compassionate care, especially from nurses; and the practical benefits of care, such as no cost and access to advanced equipment. Participants were mostly satisfied with the specialist care that they received. They were also satisfied with the diagnostic and treatment processes and believed that the staff they met were well-qualified and experienced, as summarized by the statement*: “Everything I experienced was positive, such as attention, good treatment, and care.” (Participant 2).*

As a participant described, satisfaction was also linked to their relationship with the health-care staff and the medical equipment used.

*The diagnostic process and treatment are both very good here. I was diagnosed correctly, and I received proper treatment .... I got the help I needed, and I am completely satisfied with that. (Participant 4).*

Most participants described the care received from nurses using terms that portrayed a sense of compassion. They often used the words *“kind”* and *“caring”* to describe this. For example:

*I am very satisfied with the help I got. Honestly, they overwhelmed me with their care and kindness. (Participant 9).*

*Yes, I got the help I needed, and I am very satisfied with the help I got. They were very kind and caring. (Participant 10).*

Similarly, some participants described how the health-care staff in Norway were kind and compassionate by using phrases such as *“showed me humanity”.* One participant mentioned *“I was treated with full respect and humanity. I was embarrassed getting so much help, they were helping me with everything.” (Participant 17).*

Another participant compared Norway to other countries: *“I was treated with full respect. I was hospitalized in many different countries. I can’t compare Norway to the other countries related to respect and how they deal with the patient.” (Participant 18).* This level of compassion was not often experienced in treatment in their home countries, and it could, for some participants, be a reason for why they expressed a preference for the Norwegian health-care system.

The participants experienced respect for their religious needs. Participants who wanted to pray felt comfortable practicing their religion in the hospitals.

*Yes, I used to pray in the hospital. Once, the doctor and nurse entered the room while I was praying, they left and came back after a while, and they asked me about the prayer times so that they wouldn’t bother me while I am praying. I was very pleased with that. (Participant 2).*

Although participants expressed general satisfaction with the diagnosis and treatment they received in Norway, it was clear that many participants felt that the doctors in their home countries were more qualified and experienced and therefore had more specialist knowledge of health conditions.

*I would like to get treatment in my country. The doctors there have more experience. But the equipment here is much better. Also, the nursing staff is better here, they don’t complain, and they are doing a great job. (Participant 17)*

The participants believed that Norway had more advanced equipment, which was more accessible due to the welfare-based health care. Hence, they preferred the Norwegian health-care system compared with those in their home countries because of the former being more advanced in terms of medical equipment and having more compassionate staff and respect for the dignity of patients.

## Discussion

The present study focused on how hospitalized Arabic-speaking patients experienced their interaction with the Norwegian health-care system. The interviews revealed that the patients experienced challenges of understanding and being understood, and they missed the holistic and direct care that they received in their home countries, but they also experienced certain positive aspects of the Norwegian health-care system. The study showed the importance of religious and cultural norms that influence how individuals experience illness, what they consider is needed when they are sick, and the outcome of the treatment received. The different expectations the patient and their families have of the health-care provider have an impact on how they experience this stressful time.

Participants who had a satisfactory language proficiency described good communication with health-care providers, as opposed to those with limited language proficiency. Language proficiency can have an impact on the way patients with language barriers report their symptoms to their family members who might act as interpreters. This situation can be confusing for health-care providers; these findings are similar to those of several other studies [26, 37].

The present study also confirmed the importance of communication and professional interpreters’ role in client satisfaction at hospitals under investigation. This finding might also suggest that the perception of miscommunication is exaggerated when it is linked with a perception of wrong diagnosis or procedural mistakes and delays. This is consistent with a previous report that demonstrated the importance of communication between service consumers and providers [23].

Furthermore, it was found that the themes of cultural communication and professional interpretation acquired their importance because of the underlying and latent theme of satisfaction with health care and diagnosis of disease. The feeling of disease aggravation and dissatisfaction was almost linked with a communication gap between the patients and health care providers. This finding is consistent with previous findings that a communication gap can amplify the perception of illness and anxiety [38, 39].

We also found that the cross-cultural situation of hospitalization creates some difficulties in internalizing the role expected of patients who belong to a culturally distinct background [40]. This may also be the case for those patients who had not been assimilated into the Norwegian culture because of the short duration of their stay in Norway [41]. A Norwegian survey showed low health-literacy among immigrants, and this is challenging in their interaction with the health care services [42]. The phenomenon of dissatisfaction was characterized by delayed appointments, indirect access to health care with perceptions of aloofness and nonparticipation, issues with interpretation of medical terminologies, and lack of cross-cultural competence. This finding confirms a similar study of Syrian refugee experiences in Norway [43].

Interpreters with a medical background were perceived to be more professional than nonmedical interpreters [44, 45]. A previous study demonstrated that culture plays an important role with regard to foreign patients’ attitudes toward critical issues during hospitalization, such as death and dying in the intensive care unit, an inspection of genitals by a physician of the opposite sex, the role of religion, presence of family, and use of alternative healing practices [13]. Moreover, cultural competence plays an important role throughout the utilization of health-care services [46].

Furthermore, the participants were generally satisfied with the quality of food and their ability to practice their faith in the hospitals. The participants who displayed limited trust in the system largely tended to display their dissatisfaction with other facilities, such as food. Cultural differences also occurs because the Norwegian system relies on a strong bureaucratic tradition and normative practices [3], while the patients tend to have a “walk-in” approach, which is possibly linked to the private health-care systems available in their home countries. Nonetheless, there exist some issues related to the gender of the attending physician, presence of relatives in the hospital, and perception about the food, which has only tangentially been addressed in this study.

A rather important thematic zone that emerged in this study was the one that incorporates the clashing values of holism and individualism. Several studies have demonstrated the importance of cultural values in human actions and choices [47]. Moreover, non-Western immigrants from the eastern and Asian regions are likely to adhere to collectivism as a cultural value [2]. Cultural values are significant determinants of everyday behavior, and they are also a part of the nursing education syllabus. Many patients adhere to a collectivist worldview supported by holistic values [48]. This implies that disease and illness as well as healing are likely to have underlying religious connotations [49]. In fact, the Islamic scripture uses the Arabic word *Maradh (disease)* to reflect a spiritual and moral abnormality. Hence, there exists a dual conception of disease in the minds of the Arabic-speaking patients, and it is believed that a fully healed patient will receive good health as God’s mercy. This was also indicated in the repetitive use of the term “God” for the doctor by the patients. The presence of family and praying at the hospital is part of this conception of disease, and behaviors such as crying and supplicating via prayers for good health and miraculous healing are considered normal. These findings are consistent with those reported previously on the role of families within the health-care system [50].

**Fig. 2:**
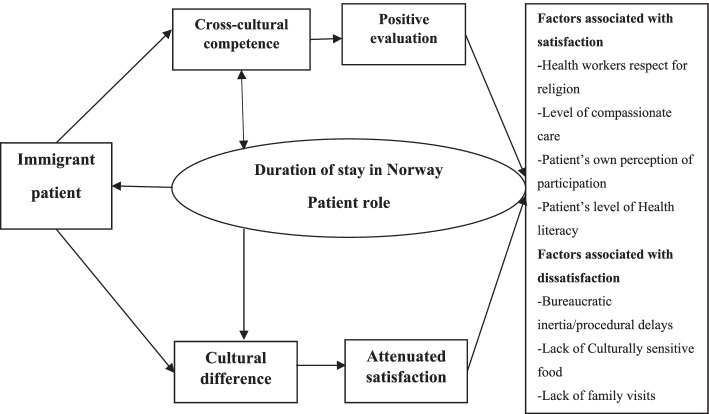
A model for foreign patients’ satisfaction

Figure 2 shows how immigrant patients, the durations of their stay in Norway, and the consequent cross-cultural and lingual competence play a direct role in the favorable evaluation of the Norwegian health-care system. Furthermore, cultural, and lingual differences, interpreted as difficulties in performing the role of patients, are a good predictor of the difficulties faced by patients and their negative evaluation of the health-care system. The factor with the highest correlation of patient’s reported level of satisfaction with the health system/visit, was duration of stay in Norway. This finding requires further quantitative research to validate the assumptions generated in this study.

### Strengths and limitations

A limitation of the present study is that it included only two female patients. This might be due to the scarce availability and willingness of female patients to undergo in-depth interviews with a male researcher. In addition, most Arab immigrants coming to Norway in the last 10 years have been male [51]. Another rather less significant limitation is that just one of the authors is a native Arabic-speaker and performed the interviews translation. However, all the authors have command over English language to decide about the degree of verisimilar patterns of narratives due to their existing familiarity with the literature.

The strengths of this study are that the participants had experiences from five different hospitals and the interviews were performed in Arabic, which was the participants’ mother tongue. All researchers are sufficiently knowledgeable about the difference in the health system in Norway and other countries.

## Conclusion and recommendations

Some specific recommendations for the health-care system are as follows. Newcomers should be given information about the health-care system in Norway and how it works. The use of a specialized interpreter service that entails the engagement of medical professionals or nursing staff, even those who are completing their education, appears to be valuable. Similarly, having a pool of visual materials to facilitate the understanding of complex medical procedures will alleviate the burden of interpreting in a culturally sensitive manner. Launching and promoting health-care-centric cultural awareness for the patients is also recommended. It is also desirable to educate nursing staff about the norms of gender, food, and religion. Finally, allowing relatives inside the ward to provide emotional and physical support to the patients is an idea worthy of being implemented. Further quantitative investigations are required to establish the association between the cultural competence of patients and its predictors, such as linguistic competence and health-system literacy.

The present study focused on the experiences of Arabic-speaking patients regarding their interactions within the Norwegian health-care system. The findings showed that the patients had some linguistic problems in understanding the doctor and being understood. Hence, as suggested by the participants, a better interpretation of technical and sensitive terminologies requires the engagement of professional interpreters. Similarly, some instances of using graphics and visual aid by the doctor had proven to be a valuable practice. Some patients did feel uncomfortable about the food; hence, the ready availability of halal food would be a good alternative to vegetarian food.

Most participants showed a very positive attitude toward the provision of compassionate care, advanced equipment, and respect for diversity and religion in the Norwegian health-care system. Nonetheless, the patients felt negatively toward delays in the provision of care services and suffered because of cultural differences. Hence, they had difficulty becoming accustomed to a patient role that was different from the one in their home country. Based on the nexus between the socialization in their home country and the cultural differences in Norway, we can hypothesize that cultural integration and competence are important predictors of foreign patients’ satisfaction with the Norwegian health-care system.

## Data Availability

The datasets used and/or analyzed during the current study are not publicly available due to General Data Protection Regulation laws but are available from the corresponding author on reasonable request and with permission from the Norwegian Centre for Research Data.

## Abbreviations

GP: General physician
CAM: Complementary and alternative medicine
